# The Impact of Short-Form Video Addiction on Sleep Quality: The Mediating Roles of Emotional, Cognitive, and Executive Components

**DOI:** 10.3390/bs16071232

**Published:** 2026-07-20

**Authors:** Shiying Yang, Tao Dong, Xiaoyan Wang, Shaobo Lyu, Bo Song

**Affiliations:** 1 Hebei Key Laboratory of Mental Health and Brain Science, School of Psychology and Mental Health, North China University of Science and Technology, Tangshan 063000, China; psyyangshiying@163.com (S.Y.); 18222206613@163.com (T.D.); 2 Library, North China University of Science and Technology, Tangshan 063000, China; xiaoyan_903@126.com; 3 Student Affairs Office, North China University of Science and Technology, Tangshan 063000, China

**Keywords:** short-form video addiction, sleep quality, social anxiety, self-control, I-PACE model, serial mediation

## Abstract

With the rapid growth in the number of short-form video users, short-form video addiction (SVA) has drawn increasing attention due to its significant impact on individuals’ daily lives. This study examines the relationship between short-form video addiction and sleep quality, as well as the mediating mechanisms of social anxiety and self-control. Using the College Student Short-Form Video Addiction Scale, the Interaction Anxiousness Scale, the Brief Self-Control Scale, and the Pittsburgh Sleep Quality Index, we surveyed 677 Chinese college students. Correlation and mediation analyses were conducted using SPSS and the PROCESS macro. The findings revealed that SVA was negatively associated with sleep quality both directly and indirectly, with higher social anxiety and lower self-control mediating the indirect effect. By showing that addictive behavior is linked to its risk factors through affective, cognitive, and executive pathways, this study provides preliminarily elucidates how addictive behavior feeds back to reinforce its own risk factors proposed in the Interaction of Person–Affect–Cognition–Execution (I-PACE) model.

## 1. Introduction

With the rapid growth of internet users and the widespread adoption of smartphones, patterns of internet use and engagement have undergone profound changes, leading to the rapid development of a new form of digital content sharing—short-form video (SFV). SFV are video content ranging from a few seconds to a few minutes in length. Due to their ease of viewing, diverse formats, and high user appeal, they are currently the most popular type of video product (Feng, 2024; Huangfu et al., 2022; Y. Wu et al., 2021). Since the launch of the SFV platform TikTok in 2016, its short content, user-friendly interface, and highly effective recommendation algorithm have propelled it to rapid prominence over the past decade, making it a mainstream method of internet content dissemination (T. Li et al., 2025). Reports indicate that by 2025, TikTok had 1.59 billion users, representing 27.5% of the global population aged 18 and older, and this figure continues to grow (DataReportal, 2025). Furthermore, as SFV have become increasingly popular, other major platforms such as YouTube and Instagram have integrated similar features to meet user demand. Adolescence and early adulthood are critical periods for the development of intimate relationships, during which individuals tend to use social media more frequently. Reports indicate that approximately 20% of Chinese adolescents frequently watch short videos (Brautsch et al., 2023; R. Liu, 2022).

Recommendation algorithms are a key feature of short-video platforms; through these algorithms, platforms can accurately identify user preferences and continuously provide engaging content (Marengo et al., 2021; Y. Yang et al., 2024). In this immersive, infinite-scroll viewing experience, the lack of natural stopping points makes it difficult for users to stop watching short videos on their own (Kim & Kim, 2024). Frequent reinforcement, rapid content delivery, and algorithm-driven recommendation systems all contribute to users easily developing short-form video addiction (SVA) (A. Wu & Yao, 2026). SVA refers to a user’s uncontrollable urge to watch SFV, leading to significant behavioral dysfunction or attention deficits, which in turn result in difficulties in interpersonal relationships, academic performance, work, and other areas of functioning (Liao, 2024). Existing research indicates that short video usage is closely linked to a decline in users’ mental health; users who consume short videos experience higher levels of stress and are more likely to develop anxiety or depression (Nguyen et al., 2025; C. Zhu et al., 2024). Additionally, SVA is associated with a decline in cognitive abilities, such as attention, and has a negative association with interpersonal interactions in real-world social settings (T. Li et al., 2025; Zhai et al., 2024). Adolescence is a critical stage of physical and mental development, during which the cerebral cortex undergoes extensive synaptic remodeling and is more susceptible to interference from various risk factors, potentially affecting lifelong development (Lockhart et al., 2018). Excessive use of social media, such as short videos, may affect the normal brain development of adolescents and impair their attention, memory, and learning abilities (Zhan & Zhu, 2025). At the same time, reviews indicate that age influences the association between problematic social media use and sleep quality, with more pronounced effects among younger users. This suggests that adolescents are a high-risk group for SVA and should be a focus of attention (Chen et al., 2026).

With the continuous growth of the short video user base, its potential negative association with adolescents’ well-being is increasingly drawing scholarly attention. Despite accumulating evidence linking SFV use to poor sleep quality, several research gaps remain. First, most studies have focused on direct associations, whereas the specific psychological mechanisms—particularly those involving multiple interacting pathways—remain incompletely understood. Second, although social anxiety and self-control have been separately examined as mediators in related contexts (L. Jiang & Yoo, 2024; Krishnan & Chew, 2024), their interplay has not been simultaneously investigated within a unified theoretical framework. Third, while the I-PACE model posits that addictive behaviors and risk factors constitute a vicious cycle, the specific pathways through which addictive behaviors negatively reinforce or exacerbate their own risk factors remain underexplored (Brand et al., 2016, 2019). To address these gaps, the present study, guided by the I-PACE model as an organizing framework, tested a serial mediation model in which SVA is associated with sleep quality through heightened social anxiety (an affective–cognitive component) and diminished self-control (an executive component). By elucidating how short-form video addiction is bidirectionally linked to risk factors through the interplay of these components, this study extends our understanding of the reverse pathway reinforcement processes specified in the I-PACE model.

## 2. Literature Review and Theoretical Assumptions

The I-PACE model was proposed by Brand et al. in 2016 and is primarily used to explain the mechanisms underlying the development and maintenance of internet-related addictions (Brand et al., 2016). Following its refinement and expansion in 2019, the model can now broadly explain various addictive behaviors (Brand et al., 2019). This theory posits that the development of addictive behavior primarily involves four components: Person, Affect, Cognition, and Execution. Here, *p* represents individual susceptibility factors, such as personality traits and motivations for use; A&C refer to emotional and cognitive responses to external stimuli, such as social anxiety and rumination; and E denotes an individual’s executive functions, such as inhibitory control and decision-making. The P factor does not directly trigger addictive behaviors; instead, it influences behavior indirectly by shaping cognitive patterns, modulating emotional responses, and weakening inhibitory control, while the A, C, and E components also interact with one another. As short-form video use gratifies users’ needs, the behavior is repeated and may progress to addiction. Critically, addictive behaviors, in turn, reinforce maladaptive cognitive styles and short-form-video-related cognitive biases, thereby exerting a backward effect on the P factor. This bidirectional relationship forms a self-perpetuating vicious-cycle. As addiction advances, the dominant motivation for short-form video use shifts from seeking positive affect to avoiding negative affect, indicating a transition from positive to negative reinforcement. During this stage, control over use markedly declines, and the vicious-cycle accelerates, becoming progressively harder to break and ultimately leading to more severe negative outcomes.

### 2.1. SVA and Sleep Quality

Sleep quality is a concept used to assess the quality of an individual’s sleep. Buysse (2014) categorized sleep quality into six dimensions to integrate subjective evaluations and objective indicators of sleep quality. Sleep is one of the most fundamental physiological needs of humans, and sleep quality directly impacts an individual’s physical and mental health. Adequate sleep not only promotes physical development and improves the immune system but also regulates emotional states (Besedovsky et al., 2019). Conversely, reduced sleep quality may negatively impact an individual’s emotions, cognition, and willpower, and in severe cases, lead to emotional disorders such as anxiety and depression (Dai et al., 2026; Spytska, 2024). It is evident that sleep is crucial for an individual’s physical and mental health; however, relevant studies indicate that sleep quality among young people is declining, with sleep problems being particularly common among college students (F. Wang & Bíró, 2021). This phenomenon may be related to the high rate of SFV usage among college students. Research indicates that adolescents tend to watch short videos in the evening before bedtime. The blue light emitted by electronic devices and the use of mobile devices at night can disrupt melatonin secretion, which can lead to difficulty falling asleep at night and reduced sleep quality (Haripriya et al., 2018; Wahl et al., 2019; B. Yang et al., 2026). Other studies have shown that SFV use alters users’ pre-sleep cognitive arousal, thereby reducing sleep quality (K. Wang & Scherr, 2022). On the other hand, according to the time-displacement theory, time spent on short videos reduces the time available for other beneficial activities; adolescents’ addiction to short videos may encroach on sleep time, thereby lowering sleep quality (Cain & Gradisar, 2010; X. Zhu et al., 2023). According to the I-PACE model, addicted SFV users experience impaired executive function when exposed to short video cues, leading them to prioritize short-term gratification while neglecting longer-term negative consequences (Brand et al., 2016). This may be associated with them delaying sleep in exchange for the immediate satisfaction provided by short videos, resulting in insufficient sleep duration and reduced sleep quality. Numerous empirical studies have shown a negative correlation between SFV usage and sleep quality. Short video usage is associated with reduced sleep duration, prolonged sleep latency, difficulty falling asleep, early awakening, and daytime fatigue (Bilali et al., 2025; Chao et al., 2023; L. Jiang & Yoo, 2024; Nguyen et al., 2025; Xiao et al., 2026). However, the relationship between SVA and sleep quality is not unidirectional; relevant longitudinal studies indicate that lower sleep quality is associated with higher levels of SVA, meaning that sleep quality is also a risk factor for SVA (Oh & Heo, 2025; D. Wang et al., 2025). Therefore, this study proposes the following hypothesis:

**H1.**  *SVA is negatively associated with sleep quality.*

### 2.2. The Mediating Role of Social Anxiety

Social anxiety refers to an irrational state of fear and anxiety experienced by individuals in social situations due to a fear of negative evaluation by others, often accompanied by social avoidance behaviors (Alvi et al., 2022). In the I-PACE model, social anxiety is a key emotional and cognitive response. Addictive behaviors, in turn, may reinforce pre-existing emotional and cognitive biases through reinforcement via gratification or compensatory effects, thereby being associated with a vicious cycle (Brand et al., 2016). Addictive behavior may primarily be associated with social anxiety through two pathways, thereby being indirectly linked to sleep quality. On the one hand, according to the time-displacement theory, time spent on social media reduces time allocated to real-world social activities, leading to social anxiety in real life (Cain & Gradisar, 2010; L. Jiang & Yoo, 2024). On the other hand, according to the social comparison theory, through short videos, users are exposed to others who are superior to them in certain aspects, leading to upward comparison; this sense of inadequacy triggers negative emotional states such as inferiority and anxiety (Festinger, 1954). Short videos on TikTok attempt to construct ideal role models with perfect appearances and lifestyles, and users’ upward comparison with these idealized role models is associated with significantly reduced life satisfaction and heightened social anxiety (Pruett, 2024; Weber et al., 2025). The Attentional Control Theory posits that individuals with social anxiety experience persistent worry and excessive rumination, and are more prone to repetitive negative thinking before bedtime, leading to difficulty falling asleep, sleep disruption, and reduced sleep quality (Eysenck et al., 2007; Richardson et al., 2025; Zhang et al., 2024). A substantial body of research supports the notion that SVA is associated with social anxiety, which in turn is associated with reduced sleep duration and lower sleep quality among adolescents (Du et al., 2026; L. Jiang & Yoo, 2024; Zhai et al., 2024). Based on this, the present study proposes the following hypothesis:

**H2.**  *Social anxiety mediates the relationship between SVA and sleep quality.*

### 2.3. The Mediating Role of Self-Control

Self-control is the ability to change one’s own thoughts, emotions, and behaviors, or to inhibit impulses and habits (Maranges & Baumeister, 2016). Self-control is a crucial ability that plays a significant role in an individual’s physical and mental health and development, and can also enhance life satisfaction and well-being (de Ridder, 2024). High levels of self-control can improve children’s social skills and academic performance, while reducing the risk of depression, delinquency, and illicit drug use among adolescents (Robson et al., 2020). A large body of prior research has indicated that SVA is associated with low levels of self-control. Furthermore, some scholars have found that SVA is significantly negatively correlated with prefrontal executive function (Mona et al., 2026; Yan et al., 2024). The dual-system model of self-control posits that an individual’s cognitive and behavioral control is primarily driven by two systems: the impulsive system and the reflective system (Hofmann et al., 2009). The impulsive system operates automatically, is driven by emotions, and tends to seek immediate rewards, whereas the reflective system relies on self-control to exert conscious regulation. Under normal circumstances, individuals use the reflective system to achieve long-term goals in a goal-oriented manner; however, prolonged use of short videos alters users’ behavioral patterns, making them more inclined to rely on the impulsive system (T. Jiang et al., 2025). As the reflective system is no longer activated, activity in brain regions dominated by the prefrontal cortex is suppressed, leading to a sustained decline in individuals’ self-control (Su et al., 2023). When self-control declines, adolescents are unable to stop using their phones before bedtime, leading to delayed sleep onset, disrupted sleep–wake rhythms, and reduced sleep duration, which in turn is associated with lower overall sleep quality (Buysse, 2014; Guarana et al., 2021; Ling et al., 2024). Based on this, the present study proposes the following hypothesis:

**H3.**  *Self-control mediates the relationship between SVA and sleep quality.*

### 2.4. The Serial Mediating Role of Social Anxiety and Self-Control

Social anxiety and self-control are key mediating factors in the relationship between SVA and sleep quality. According to the Limited Resource Model of Self-Control, self-control relies on a finite and depletable psychological resource; if this resource is insufficient, individuals cannot effectively regulate their behavior (Baumeister et al., 1998). The Cognitive Interference Theory suggests that social anxiety consumes a significant amount of an individual’s cognitive resources, making it difficult to exercise self-control and maintain regular sleep schedules and duration, thereby reducing sleep quality (Blackhart et al., 2015; Northern, 2010). Research indicates that high levels of social anxiety may be associated with reduced individuals from activating prospective control, leading to a decline in inhibitory executive function. According to the I-PACE theory, emotional and cognitive responses do not operate independently of executive function but rather interact with it, jointly driving negative behaviors. Consequently, social anxiety is associated with sleep quality through its interference with self-control (Brand et al., 2016). Additionally, the I-PACE model posits that addictive behaviors create a negative cycle by exerting a reverse influence on other components; thus, SVA may be indirectly associated with sleep quality through emotional, cognitive, and executive components. Based on this, the present study proposes the following hypothesis:

**H4.**  *Social anxiety and self-control play a serial mediating role in the relationship between SVA and sleep quality.*

**Figure 1:**
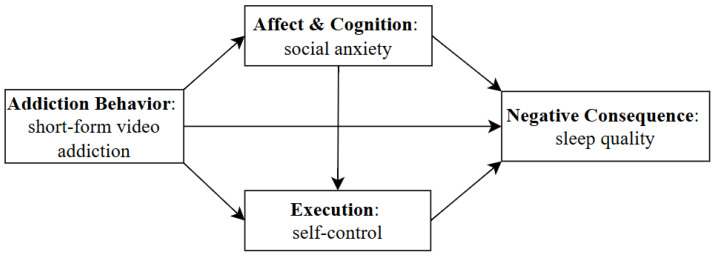
Hypothesised model diagram.

The conceptual model depicting these hypothesized relationships is presented in Figure 1.

## 3. Materials and Methods

### 3.1. Study Population

According to the principle of multiple factor analysis, the minimum sample size should be 5 to 10 times the total number of questionnaire items. To account for potential participant attrition and other issues, a convenience sampling method was adopted. We aimed to collect a minimum of 500 valid responses to ensure the representativeness and reliability of the study’s findings.

This study conducted a survey in June 2025 among students from a university in Tangshan City, Hebei Province, China. Prior to completing the questionnaire, the purpose of the investigation was explained to the students, and their informed consent was obtained. The questionnaires were primarily distributed and collected using an online platform during public classes. Upon completion of data collection, a rigorous screening process was implemented. Questionnaires with excessively short completion times or those with incorrect responses (e.g., incorrectly filled sleep onset time, age, or class) were excluded to minimize the interference of invalid data with the research outcomes.

A total of 714 questionnaires were distributed, and 677 were deemed valid (299 male, 378 female), resulting in a valid response rate of 94.8%. Detailed demographic information of the participating college students is presented in Table 1. The sample was predominantly composed of second-year students (78.7%), reflecting the enrollment structure of the surveyed public courses. This concentration limits the generalizability of the findings across academic years.

### 3.2. Measurement Tools

SVA. In this study, the level of SVA was measured using the SVA Scale for College Students, developed by Qin et al. (2019). This rigorously designed scale comprises 14 items distributed across four core dimensions: Withdrawal (5 items), Escape (3 items), Loss of Control (4 items), and Inefficiency (2 items). Each item is rated on a five-point Likert scale ranging from 1 to 5, with higher total scores indicating a higher level of SVA. In the current study, the scale demonstrated good internal consistency, with a Cronbach’s α coefficient of 0.86, supporting its reliability for measuring the construct.

Interaction Anxiousness. In this study, the level of social anxiety was measured using the Interaction Anxiousness Scale (IAS) developed by Leary (1983). The scale consists of 15 items, four of which are reverse-scored. Responses are recorded on a five-point Likert scale, with higher total scores indicating a higher level of social anxiety. The IAS, known for its brevity and ease of administration, has been widely used in adult populations, including college students. Regarding its psychometric properties, the scale has been validated in numerous studies. In the present study, the IAS demonstrated good internal consistency, with a Cronbach’s α coefficient of 0.86.

Self-Control. In this study, the level of self-control was assessed using the Brief Self-Control Scale (BSCS) developed by Morean et al. (2014). This concise scale comprises a total of 7 items, which measure two dimensions: self-discipline and impulse control. Responses are recorded on a five-point Likert scale. The BSCS has demonstrated cross-cultural consistency and has been widely applied in research across various countries and cultural contexts. It has also been validated within the Chinese university student population, showing good reliability and validity. In the current sample, the BSCS exhibited good internal consistency, with a Cronbach’s α coefficient of 0.83.

Sleep Quality. In this study, the level of sleep quality was assessed using the Pittsburgh Sleep Quality Index (PSQI) developed by Buysse et al. (1989). The PSQI comprises seven key components—subjective sleep quality, sleep latency, sleep duration, habitual sleep efficiency, sleep disturbances, use of sleep medication, and daytime dysfunction—which together provide a comprehensive assessment of an individual’s subjective sleep quality. The scale has demonstrated good reliability and structural validity and has shown high stability and cross-cultural applicability. For this study, we employed its 18 self-rated items for evaluation. Each PSQI item is scored on a scale of 0 to 3, with the global score ranging from 0 to 21. A higher total score indicates poorer sleep quality. In the current sample, the PSQI demonstrated excellent internal consistency, with a Cronbach’s α coefficient of 0.71. Although the Cronbach’s α coefficient obtained in the present study was relatively low (0.71), a systematic review has noted that the internal consistency of the PSQI varies considerably across different populations (Mollayeva et al., 2016). A validation study conducted among Chinese medical university students reported a comparable Cronbach’s α of 0.734 for the PSQI (Zheng et al., 2016). This suggests that the internal consistency observed in the present sample is within an acceptable and contextually reasonable range for a Chinese college student population. The PSQI global score of >5 is widely used as a cut-off for screening poor sleep quality, whereas the validation study by Liu et al. in the Chinese population adopted a cut-off of >7 to identify clinically significant sleep problems (X. Liu et al., 1996). In the present sample, 257 participants (38.0%) scored above 5, and 119 participants (17.6%) scored above 7 on the PSQI. A previous meta-analysis reported a pooled prevalence of sleep disturbances among Chinese college students of 25.7% (95% CI: 22.5–28.9%) (L. Li et al., 2018). However, as that study did not specify the PSQI cut-off score used across the included primary studies, direct comparison with our findings is difficult. The prevalence in our sample was lower than the pooled estimate when using the >7 cut-off, but higher when using the >5 cut-off, reflecting the heterogeneity introduced by different screening thresholds.

### 3.3. Statistical Methods

The SPSS statistical package (version 23.0) was used for analysis. Categorical variables are presented as frequencies and percentages. To test the potential mediating roles of social anxiety and self-control in the relationship between SVA and sleep quality, a mediation analysis was performed using Model 6 of the PROCESS macro (Version 4.0) for SPSS developed by Hayes. In this model, SVA served as the independent variable (X), social anxiety and self-control were the mediating variables (M), and sleep quality was the dependent variable (Y). Previous studies have indicated that gender, age, and only-child status are associated with sleep quality (Lu et al., 2021; Simpong et al., 2025; H. Wang et al., 2024). Based on these findings and the observed correlations among the variables in the present sample, we included gender, age, only-child status, and residence type as covariates in the analyses. To avoid multicollinearity, only age (rather than both age and academic year) was entered into the regression models, given the high correlation between these two time-related variables. This analysis allowed for the estimation of the indirect effects of social anxiety and self-control in mediating the relationship between SVA and sleep quality.

The mediation hypothesis was tested using the bias-corrected bootstrapping method with 5000 resamples. Ninety-five percent confidence intervals were calculated. Effects were considered statistically significant if the confidence intervals did not include zero.

## 4. Results

### 4.1. Common Method Bias

The potential issue of common method bias was assessed using Harman’s single-factor test. An exploratory factor analysis was conducted on all items from the scales measuring SVA (including its subscales), social anxiety, self-control, and sleep quality. The results revealed 10 factors with eigenvalues greater than 1. The first factor explained 22% of the total variance, which is below the critical threshold of 40%. This indicates that common method bias was within an acceptable range in the present study.

### 4.2. Correlation Analysis of SVA, Social Anxiety, Self-Control, and Sleep Quality

According to the findings in Table 2, a significant positive correlation was found between SVA and social anxiety (*r* = 0.37, *p* < 0.001). Conversely, SVA was negatively correlated with self-control (*r* = −0.39, *p* < 0.001). Furthermore, a significant positive correlation was observed between SVA and poorer sleep quality, as indicated by higher PSQI scores (*r* = 0.24, *p* < 0.001).

Social anxiety and self-control were significantly negatively correlated (*r* = −0.35, *p* < 0.001). Additionally, social anxiety was positively correlated with poorer sleep quality (*r* = 0.32, *p* < 0.001), while self-control was negatively correlated with poorer sleep quality (*r* = −0.27, *p* < 0.001).

In summary, significant correlations exist among all variables. Specifically, the results suggest that both social anxiety and self-control may play influential roles in the relationship between SVA and sleep quality, providing a preliminary foundation for the subsequent mediation effect analysis.

### 4.3. Serial Mediation Effect Test for Social Anxiety and Self-Control

A bootstrap mediation effect analysis was conducted using Model 6 of the PROCESS macro (Version 4.0) for SPSS developed by Hayes. SVA was entered as the independent variable (X), social anxiety and self-control as the serial mediating variables (M1 and M2, respectively), and sleep quality (PSQI score) as the dependent variable (Y). All demographic covariates (gender, age, only child status, and residence type) were included in the analysis. The significance of the indirect effects was tested using the bias-corrected bootstrapping method with 5000 resamples. Ninety-five percent confidence intervals were calculated.

**Figure 2:**
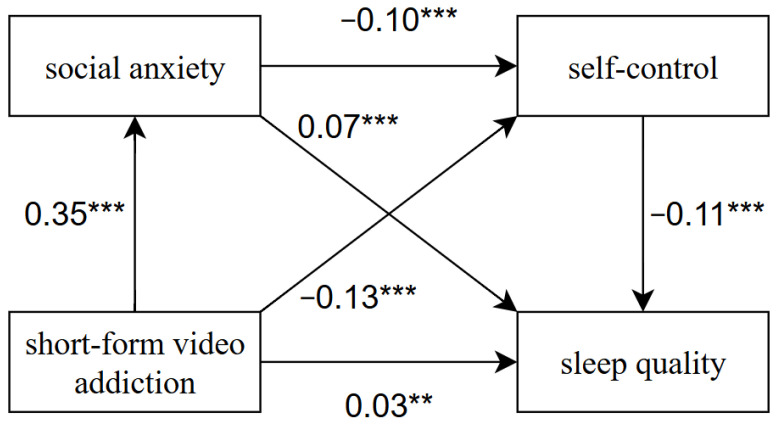
Serial mediation model of social anxiety and self-control. ** *p* < 0.01, *** *p* < 0.001.

The results of the regression analysis (see Table 3 and Table 4) and the data presented in Figure 2 indicate that all three hypothesised indirect paths are statistically significant, supporting the serial mediation model. Specifically, short-form video addiction significantly predicted social anxiety (*B* = 0.354, *t* = 10.102, *p* < 0.001) and directly, negatively predicted self-control (*B* = −0.128, *t* = −8.175, *p* < 0.001). When both short-form video addiction and social anxiety were included as predictors, social anxiety significantly and negatively predicted self-control (*B* = −0.101, *t* = −6.267, *p* < 0.001). In the final regression model predicting sleep quality, short-form video addiction (*B* = 0.032, *t* = 2.555, *p* < 0.05) and social anxiety (*B* = 0.074, *t* = 5.969, *p* < 0.001) were significant positive predictors, while self-control was a significant negative predictor (*B* = −0.113, *t* = −3.882, *p* < 0.001).

The bootstrapping analysis confirmed the significance of the indirect effects. Short-form video addiction exerted a significant indirect effect on sleep quality through: the independent mediating role of social anxiety (indirect effect = 0.026, 95% CI [0.017, 0.036]), accounting for 34.5% of the total effect (0.076); the independent mediating role of self-control (indirect effect = 0.014, 95% CI [0.007, 0.023]), accounting for 18.9% of the total effect; and the serial mediation of social anxiety and self-control in sequence (indirect effect = 0.004, 95% CI [0.002, 0.007]), accounting for 5.2% of the total effect.

These results support the existence of a serial mediation pathway. Specifically, SVA not only directly predicts higher social anxiety and poorer sleep quality but also indirectly impairs sleep quality through a sequential pathway: higher levels of SVA are associated with increased social anxiety, which in turn is associated with reduced self-control, ultimately leading to more severe sleep problems.

## 5. Discussion

Based on the I-PACE model, this study examined the mechanism linking SVA to sleep quality among college students, with a focus on the mediating roles of social anxiety and self-control. The results supported all four hypotheses: SVA was not only directly and negatively associated with sleep quality (H1) but also indirectly associated with sleep quality through the independent mediating effects of social anxiety (H2) and self-control (H3), as well as through a sequential mediating effect (H4). Notably, the total indirect effect accounted for 58.7% of the total effect, indicating that the SVA–sleep quality association was largely carried by psychological mechanisms involving affective–cognitive and executive components.

The direct effect of SVA on sleep quality was statistically significant but relatively small in magnitude. This finding is consistent with previous research on problematic mobile phone use and social media use, which also reported a direct negative association with sleep quality (Ahmed et al., 2024; Kumar et al., 2025; Waheed et al., 2024). The theoretical mechanisms underlying this direct pathway are well established. On the one hand, the time displacement hypothesis suggests that immersion in SFV encroaches on sleep time, leading to delayed bedtimes and shortened sleep duration (Cain & Gradisar, 2010; Zhao & Kou, 2024). On the other hand, the interactive and algorithm-driven nature of SFV can induce pre-sleep cognitive arousal, while blue light emitted by smartphone screens suppresses melatonin secretion; together, these factors contribute to difficulty falling asleep among college students (Cain & Gradisar, 2010; Haripriya et al., 2018; Wahl et al., 2019; K. Wang & Scherr, 2022). The finding that the direct effect accounted for less than half of the total effect suggests that psychological components—affective, cognitive, and executive—also play an important role in the relationship between SVA and sleep quality.

The total indirect effect represented 58.7% of the total effect, highlighting the crucial role of psychological pathways in linking SVA to poor sleep quality. This proportion is notably higher than that reported in some single-mediator studies. A study examining social anxiety as a mediator between SVA and sleep quality reported an indirect effect of approximately 37.5%, considerably lower than the 58.7% found in the present study (L. Jiang & Yoo, 2024). However, the current effect pattern aligns more closely with studies employing a sequential mediation design. For instance, research investigating the influence of physical exercise on SVA through self-control and social anxiety found a total indirect effect of 57.37% and a sequential mediation proportion of 6.24%, which are highly consistent with our findings (G. Wang et al., 2025). The relatively high proportion of indirect effect observed here may be attributable to our simultaneous inclusion of both affective–cognitive (social anxiety) and executive function (self-control) mediators, thereby capturing a more complete set of pathways. In a general college student sample, the finding that psychological mediators accounted for over 50% of the total effect suggests that a vicious cycle between addiction and sleep problems may have already begun to form, and could thus be regarded as an early warning sign even in the absence of a clinical diagnosis.

The independent mediating effect of social anxiety was the most prominent among all indirect pathways, underscoring the central role of affective–cognitive processes in the SVA–sleep quality link. SFV continuously expose users to idealized lifestyles and carefully curated self-presentations, which can easily trigger upward social comparisons and feelings of inferiority (Festinger, 1954; Pruett, 2024; Weber et al., 2025). College students operate in a relatively closed social environment and are at a developmental stage characterized by heightened sensitivity to peer evaluation, making them particularly susceptible to the social anxiety evoked by such upward comparisons. According to the attentional control theory, individuals with high social anxiety tend to experience excessive pre-sleep rumination and repetitive negative thinking; this cognitive hyperarousal makes it difficult to disengage from worry and transition into sleep, ultimately impairing sleep quality (Eysenck et al., 2007; Richardson et al., 2025; Zhang et al., 2024). Thus, the disruption of sleep by SVA goes beyond merely delaying sleep onset. Even after putting down the phone, concerns about social evaluation triggered by SFV content persist, prolonging the time required to fall asleep and reducing sleep quality.

The independent mediating effect of self-control, though smaller than that of social anxiety, was also significant. This pathway reflects the impairment of executive function that accompanies addictive behaviors. According to the dual-systems model of self-control, prolonged exposure to immediately rewarding stimuli (e.g., algorithmically recommended SFV) strengthens the impulsive system while inhibiting the reflective system (Hofmann et al., 2009). Neuroimaging evidence suggests that viewing SFV is associated with suppressed activity in the prefrontal cortex, a region that serves as the neural substrate for self-regulatory control (Su et al., 2023). As self-regulatory resources become progressively depleted, individuals’ capacity to inhibit impulses declines, leading to bedtime procrastination (Buysse, 2014; Guarana et al., 2021). This pathway reveals that SVA is linked not only to shorter sleep duration but also to a potential erosion of the psychological executive functions necessary for maintaining healthy sleep regularity.

The finding that the independent mediating effect of social anxiety was larger than that of self-control may reflect the stage of addiction represented by our sample. The I-PACE model posits that in the early stages of addictive behavior, affective–cognitive components are the primary driving forces, whereas significant impairment of executive functions typically emerges in later stages (Brand et al., 2016, 2019). The participants in this study were general college students, and their overall level of addiction likely remains in the early-to-mid stage. Accordingly, the pathway through social anxiety was more pronounced, while self-control was not yet markedly depleted. This interpretation carries important implications: as addiction deepens, the self-control pathway may become stronger and could eventually become the dominant mediating mechanism. Thus, the observed difference in mediating effect sizes is not merely a statistical artifact but may reflect clinically meaningful stage characteristics in the progression of addiction.

The sequential mediation effect, though small in magnitude, reached statistical significance. It should be recognized that any sequential mediation effect is the product of multiple regression coefficients and is therefore inherently smaller than independent mediation effects. The fact that this pathway remained statistically significant despite this constraint attests to its robustness. The theoretical basis for this pathway draws on the limited resource model of self-control and cognitive interference theory (Baumeister et al., 1998; Northern, 2010). Social anxiety is characterized by persistent self-focused attention and ruminative thoughts about social evaluation, which consume the finite psychological resources required for effective self-regulation (Blackhart et al., 2015). Once these resources are depleted by anxiety, individuals find it more difficult to inhibit the urge to continue watching SFV, resulting in bedtime procrastination and, ultimately, diminished sleep quality. These findings provide empirical evidence for the reverse reinforcement loop proposed by the I-PACE model and deepen the understanding of the model.

Taken together, the differences in effect sizes among the three indirect pathways and their theoretical implications suggest the following practical interventions. First, the mediating effect of social anxiety was the most prominent (34.5%), indicating that in the early stages of addiction, interventions should prioritize affective–cognitive components. Educational institutions and public health agencies could develop programs that help users understand how recommendation algorithms construct virtual ideals of a perfect life, thereby alleviating the social anxiety arising from upward comparisons. Concurrently, emotion regulation skills can be taught to reduce the depletion of cognitive resources by social anxiety. Such efforts could effectively interrupt the vicious cycle at an early stage. Second, although the mediating effect of self-control was smaller, it should be noted that self-control is likely to be severely compromised as addiction progresses. At this stage, access to SFV platforms should be restricted, with external constraints reducing the reliance on internal inhibitory capacity. In parallel, psychological therapies can be employed to restore individuals’ depleted self-control resources. Finally, at the institutional level, given that the recommendation algorithms of SFV platforms are designed to maximize user retention and are inherently highly addictive, policy interventions are warranted. These may include mandating algorithmic transparency, setting default time limits for young users, or restricting harmful content (Donadoni et al., 2025; Hilbert et al., 2024; Merrill et al., 2025; Roberts & David, 2025). Such measures would help to fundamentally reduce the risk of SVA.

Several limitations of the present study should be acknowledged. 1. All measures relied on self-report instruments, which may introduce common method bias. Although Harman’s single-factor test showed that the first factor explained only 22% of the total variance, well below the 40% threshold, it must be noted that this test has limited sensitivity to moderate method bias. Future research could address this concern by incorporating objective data, such as objective measures of SFV use time and sleep duration. 2. The sample was drawn from a single university in northern China through convenience sampling, which may limit the generalizability of the findings. A recent review indicated that Eastern versus Western culture moderates the strength of the association between electronic media use and sleep problems (Han et al., 2024). Given that research on SVA has been concentrated in East Asian contexts, future studies should conduct cross-cultural validation in Western and other culturally diverse settings to examine the cultural universality or specificity of the present findings. 3. The cross-sectional design precludes rigorous causal inferences regarding the relationships among the variables. To confirm the temporal ordering of the variables proposed in this study, longitudinal data are needed in future research. 4. Although this study focused on the pathway from SVA to sleep quality, longitudinal evidence has shown that poor sleep quality is also a risk factor for addictive behaviors, representing the other half of the vicious cycle described by the I-PACE model (Oh & Heo, 2025; D. Wang et al., 2025). Our mediation model examined only the effect of addiction on sleep quality, without testing the reverse pathway from sleep quality back to addiction. Future studies should adopt longitudinal cross-lagged panel designs or ecological momentary assessment methods to fully capture the vicious cycle process described by the I-PACE model. 5. Several potential confounding variables were not measured in this study, such as chronotype, daily SFV viewing time, and stage of addiction development. Ample evidence indicates that evening-type individuals are more likely to develop SVA and tend to have poorer sleep quality (F. Wang & Bíró, 2021). The omission of these variables may affect the estimation of the true mediation effects. Future research should include these factors as covariates in the model and examine whether different mediators play distinct roles at different stages of addiction.

## 6. Conclusions

Based on the I-PACE model, this study examined the association between SVA and sleep quality among college students and its underlying psychological mechanisms. The results showed that SVA was not only directly and negatively associated with sleep quality, but also indirectly associated with sleep quality through the independent mediating effects of social anxiety and self-control, as well as through a sequential mediating effect. The total indirect effect accounted for 58.7% of the total effect, indicating that psychological mechanisms play an important role in the SVA–sleep quality link. Notably, the mediating effect of social anxiety was markedly larger than that of self-control. This difference may reflect the fact that our sample of general college students is at a relatively early stage of addiction, where addictive behavior primarily exerts its influence through affective–cognitive pathways while executive function has not yet been substantially impaired. Furthermore, although the sequential mediation effect was small in magnitude, it supports a sequential transmission mechanism from affective–cognitive to executive components. At the theoretical level, this study provides empirical support for the reverse reinforcement loop of the I-PACE model and preliminarily elucidates how addictive behavior feeds back to reinforce its own risk factors through the interplay of affective, cognitive, and executive components. At the practical level, the findings suggest that interventions should be tailored to different stages of addiction. In the early stage, cognitive–emotional interventions targeting social anxiety should be prioritized; in later stages, external strategies or intervention programs should be adopted to compensate for depleted self-control. At the institutional level, the recommendation algorithms of SFV platforms should be regulated to fundamentally reduce users’ risk of addiction. In summary, this study elucidates the SVA–sleep quality association through dual pathways involving affective–cognitive and executive functions, offers a testable pathway for the reverse reinforcement mechanism of the I-PACE model, and provides practical guidance for interventions at different stages of addiction.

## Figures and Tables

**Table 1:** Demographic variables of the participants (N = 677).

Variables	Categories	Cases	Percentage
Gender	Male	299	44.2%
	Female	378	55.8%
Age	≤18	66	9.8%
	19	230	34.0%
	20	290	42.8%
	≥21	91	13.4%
Grade	First-year	131	19.4%
	Second-year	533	78.7%
	Third-year	8	1.2%
	Fourth-year	5	0.7%
Only child	Yes	141	20.8%
	No	536	79.2%
Residence	Urban	124	18.3%
	Town	123	18.2%
	Rural	430	63.5%
PSQI	≤5	420	62.0%
	6–7	138	20.4%
	>7	119	17.6%

**Table 2:** Correlation analysis of SVA, social anxiety, self-control, and sleep quality among college students (N = 677).

Variables	1	2	3	4
SVA	1			
social anxiety	0.37 ***	1		
self-control	−0.39 ***	−0.35 ***	1	
sleep quality	0.24 ***	0.32 ***	−0.27 ***	1

*** *p* < 0.001.

**Table 3:** Regression analysis of the serial mediation model of SVA, sleep quality, social anxiety, and self-control among college students.

Regression Equation		Overall Fit Index		Regression Coefficient		
Resulting Variables	Predictor Variables	*R* ^2^	*F*	*B*	95% CI	*t*
social anxiety	SVA	0.169	27.354	0.354	[0.285, 0.423]	10.102 ***
	Age			−0.362	[−0.995, 0.271]	−1.122
	Gender			2.095	[0.750, 3.440]	3.058 **
	Only child			0.524	[−1.170, 2.217]	0.607
	Residence			1.471	[0.598, 2.343]	3.310 **
self-control	SVA	0.201	28.162	−0.128	[−0.159, −0.097]	−8.175 ***
	social anxiety			−0.101	[−0.132, −0.069]	−6.267 ***
	Age			0.008	[0.950, −0.255]	0.062
	Gender			0.334	[−0.229, 0.898]	1.165
	Only child			0.096	[−0.609, 0.801]	0.268
	Residence			−0.124	[−0.490, 0.242]	−0.663
sleep quality	SVA	0.157	17.818	0.032	[0.007, 0.056]	2.555 *
	social anxiety			0.074	[0.050, 0.099]	5.969 ***
	self-control			−0.113	[−0.170, −0.056]	−3.882 ***
	Age			0.32	[0.121, 0.518]	3.166 **
	Gender			−0.281	[−0.706, 0.143]	−1.302
	Only child			−0.403	[−0.933, 0.127]	−1.492
	Residence			0.093	[−0.182, 0.369]	0.666

* *p* < 0.05, ** *p* < 0.01, *** *p* < 0.001.

**Table 4:** Serial mediation test for social anxiety and self-control on the relationship between SVA and sleep quality.

	Effect Size	Boot SE	Bootstrap 95% CI		Proportion of Relative Effect
			Boot LICI	Boot ULCI	
Total effect	0.076	0.011	0.054	0.099	100%
SVA → sleep quality	0.032	0.012	0.007	0.056	41.40%
Total intermediary effect	0.045	0.006	0.033	0.057	58.70%
SVA → social anxiety → sleep quality	0.026	0.005	0.017	0.036	34.50%
SVA → self-control → sleep quality	0.014	0.004	0.007	0.023	18.90%
SVA → social anxiety → self-control → sleep quality	0.004	0.001	0.002	0.007	5.20%

## Data Availability

The original contributions presented in this study are included in the article. Further inquiries can be directed to the corresponding authors.

## Abbreviations

The following abbreviations are used in this manuscript:

I-PACE	The Interaction of Person-Affect-Cognition-Execution
SFV	Short-Form Video
IAS	Interaction Anxiousness Scale
BSCS	Brief Self-Control Scale
PSQI	Pittsburgh Sleep Quality Index
SVA	Short-form Video Addiction
